# Nationwide Analysis of Failure to Rescue After Liver Transplantation

**DOI:** 10.1002/wjs.70075

**Published:** 2025-09-03

**Authors:** Kamil Hanna, Peyton Seda, Brian C. Longbottom, Shengliang He, Inkyu Lee, Avery Wilson, Kenji Okumura, David Axelrod, Hassan Aziz

**Affiliations:** ^1^ Department of Surgery Stanford University Medical Center Stanford California USA; ^2^ Department of Surgery University of Iowa Carver College of Medicine Iowa City Iowa USA; ^3^ Department of Surgery Westchester Medical Center Valhalla New York USA; ^4^ Department of Surgery Massachusetts General Hospital Boston Massachusetts USA; ^5^ Department of Surgery University Hospitals Cleveland Ohio USA

## Abstract

**Introduction:**

Failure to rescue (FTR) is mortality after a major complication. FTR may be an effective quality metric in liver transplantation (LT). However, there is a paucity of nationwide data on the rates and effects of FTR on outcomes. Our study aims to determine the nationwide rate of FTR and its impact on outcomes after LT.

**Methods:**

We analyzed the 2015–2017 Nationwide Readmissions Database, including all patients with LT. Patients were stratified into terciles of average center mortality of < 1% for low (L), 1%–5.76% for intermediate (I), and > 5.76% for high (H). Postoperative complications were identified. Primary outcomes were the rate of FTR and the predictors of FTR. Multivariable regression analysis was performed.

**Results:**

A total of 12,134 patients with LT were identified at 82 centers. The sample was stratified into L: 1770 (14.6%), I: 5914 (48.7%), and H: 4450 (36.7%). The mean age was 52.2 ± 16.6 years, and 63.1% were male. Of these, 99.7% underwent deceased‐donor LT, most commonly due to alcoholic cirrhosis (31.9%), followed by metabolic steatohepatitis (20.7%). The rate of FTR was 5%, with the most common complication being renal failure at 60.6%, followed by respiratory failure at 43.1%. FTR rate differences were significant (H: 8.8% vs. I: 3.6% vs. L: 1.3%; *p* < 0.01). Multivariable logistic regression demonstrated an independent association between FTR and H (odds ratio [OR] 1.79 [1.52–1.89]). The predictors of FTR were both patient‐ and center‐related: low‐income quartile (OR 1.23 [1.11–1.39]), malnutrition (OR 1.22 [1.09–1.29]), presenting diagnosis of biliary atresia (OR 3.39 [1.95–5.93]), presenting diagnosis of acute liver failure (OR 5.01 [4.09–6.15]), Charlson Comorbidity Index [CCI] (OR 1.24 [1.18–1.31]), frailty (OR 1.58 [1.46–1.73]), LT at a low‐volume center (< 20 cases/year) (OR 1.83 [1.78–2.01]), and readmission to a different hospital (OR 2.08 [1.78–2.11]). Protective factors were LT at a metropolitan teaching hospital (OR 0.96 [0.87–0.99]), presenting a diagnosis of primary hepatic malignancy (OR 0.66 [0.52–0.86]), high‐income quartile (OR 0.74 [0.57–0.96]), disposition to rehab (OR 0.09 [0.03–0.26]), and high‐volume centers (> 50 cases/year) (OR 0.32 [0.20–0.49]).

**Conclusions:**

FTR remains a critical issue in LT, with significant variability across centers. These findings demonstrate associations, not causation, between center‐ and patient‐level factors and FTR rates. Identifying and addressing modifiable predictors of FTR presents opportunities for improving perioperative management and postoperative care.

## Graphical abstract

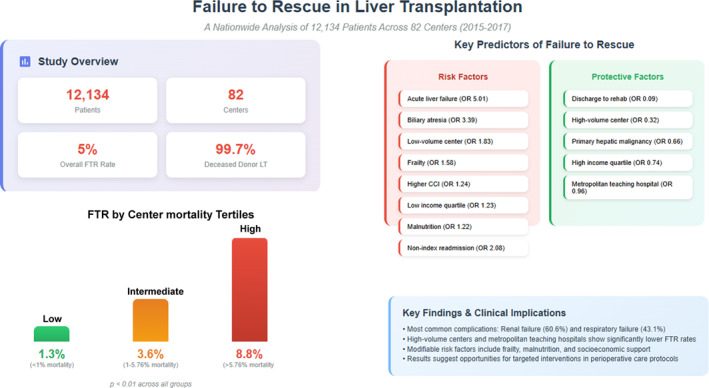

## 1 Introduction

Failure to rescue (FTR) is defined as the inability of the system to rescue patients after a major complication [1]. When it was first introduced by Silber et al. in the 1990s, it was a novel metric for delivering safe and reliable care [2]. Today, FTR is a critical quality metric to improve quality and safety, reducing mortality secondary to treatable complications [3]. The concept of FTR relies on the observation that most low‐mortality hospitals exhibit complication rates similar to those of high‐mortality centers. Despite similar complication rates, low‐mortality hospitals have lower rates of FTR, suggesting that they may better recognize and treat patients who experience complications [4].

FTR has been well defined in the surgical literature, such as in trauma, hepatobiliary resections, colorectal resections, and neurosurgery [1, 5, 6, 7]. This has resulted in a growing body of evidence identifying causes and interventions that may improve institutional FTR rates [8]. According to the Agency for Healthcare Research and Quality and the Centers for Medicare & Medicaid Services, FTR has been identified as a critical concept in patient safety and a critical part of quality improvement efforts in surgical specialties [9].

In recent times, there has been growing interest in the quality of health care delivery in liver transplantation (LT). Multiple stakeholders and regulatory bodies are interested in measuring and monitoring quality in the liver transplant process and understanding differences in quality across centers. Despite progress in donor selection and perioperative care, LT continues to be associated with significant morbidity and mortality. It has been consistently shown through research in the field that transplantation outcomes vary by transplantation center, a finding that undoubtedly contributes to low transplant success rates after transplantation [10]. Even though quality improvement efforts have attempted to narrow the mortality gap between high and low performers by reducing complication rates, there has been a growing concern that this approach may have inherent limitations because some complications are driven by patient‐related factors, which are not always preventable.

However, there is a paucity of national data on FTR rates in LT. Liver transplantation involves a highly complex postoperative course, including risks of rapid deterioration due to graft dysfunction, infection, and immunosuppression. These factors make timely recognition and management of complications critical, highlighting FTR as a meaningful quality metric for LT. Understanding the nationwide FTR rate and its effect on outcomes is crucial for optimizing care and reducing preventable mortality. This study aims to determine the nationwide rate of FTR after LT and analyze the predictors of FTR to identify potential areas for intervention. We hypothesized that high‐volume centers would have lower FTR rates due to enhanced resources and care coordination and that center‐related factors would have a stronger impact on FTR than patient‐level factors.

## 2 Methods

We retrospectively analyzed patients with LT using the 2015–2017 Nationwide Readmissions Database (NRD). The NRD is a part of the Healthcare Cost and Utilization Project (HCUP), a government‐initiated group of databases. It is the nation's largest database of all‐payer, encounter‐level hospital care data. It is developed through a federal‐state‐industry partnership and sponsored by the Agency for Healthcare Research and Quality (AHRQ) [4, 11]. The NRD is the largest all‐payer hospital care database in the United States, designed to track hospital readmissions through deidentified, linked patient data.

Hospitals are categorized by ownership, bed count, teaching status, location, and region, with a 20% probability sample collected from each category. Patient data include demographics, diagnoses, procedures, resource utilization, and outcomes. Each discharge is weighted (weight = total number of discharges from all acute care hospitals in the United States divided by the number of discharges included in the 20% sample) to ensure national representativeness. The NRD compiles up to 25 diagnoses and 15 procedures per patient using ICD‐9‐CM and ICD‐10‐CM/PCS codes and tracks patients across hospitals within a state via verified linkage numbers.

All patients undergoing LT within this period were included in this study. Patients were categorized into three groups based on average center mortality rates: low (< 1%), intermediate (1%–5.76%), and high (> 5.76%). These cutoffs were determined by the distribution of center mortality rates in our dataset, dividing centers into terciles. Although *a* < 1% cutoff may seem lower than commonly expected for LT, this threshold reflected the bottom tercile of centers in our cohort. The cutoff value for distinguishing intermediate and high mortality rates (5.76%) was determined by calculating the average mortality rate. The highest 33% of centers had mortality rates above 5.76%, whereas the lowest 33% had rates below 1%. Postoperative complications were identified, including renal failure, respiratory failure, and other significant clinical events. To eliminate the possibility of intraoperative catastrophe, patients who died within 48 h of transplantation were excluded.

The following complications were identified using ICD‐10 codes: pneumonia, respiratory failure, cardiac arrhythmias, acute congestive heart failure (CHF), cardiac ischemia, sepsis, *Clostridioides difficile* colitis, wound dehiscence, CVA, VTE, postoperative bleeding, small bowel obstruction, abscess, shock, acute kidney injury (AKI), MV > 96 h, tracheostomy, loss of independence, bile leak, rejection, GI bleed, graft dysfunction, bowel perforation, meningitis, aspergillosis, UTI, candidiasis, and vascular complications.

As a last step, failure to rescue was defined as a patient's death after at least one of the defined complications. For patients experiencing multiple complications, any postoperative death after at least one complication was classified as FTR, and all complications were included in the multivariable model to account for cumulative effects [12]. A failure‐to‐rescue rate was determined by dividing the number of deaths in patients who developed a postoperative complication by the total number of patients who experienced a postoperative complication [13].

The data contained within these specific datasets are neither identifiable nor private and thus do not meet the federal definition of “human subject.” Therefore, the institutional review board did not need to review and approve this project.

The primary outcomes assessed were the rate of FTR and its predictors. A multivariable logistic regression model was employed to evaluate independent associations between patient‐ and center‐related factors and FTR. Statistical significance was defined as *p* ≤ 0.05. The University of Iowa institutional human research review committee reviewed the project and exempted it from a review.

## 3 Results

A total of 12,134 patients with LT from 82 transplant centers were identified. The cohort was stratified into three mortality risk groups: low‐risk centers (1770 patients, 14.6%), intermediate‐risk centers (5914 patients, 48.7%), and high‐risk centers (4450 patients, 36.7%). The mean age of the cohort was 52.2 ± 16.6 years, with a predominance of male patients (63.1%). The majority (99.7%) underwent deceased‐donor LT, with the most common indications being alcoholic cirrhosis (31.9%) and metabolic steatohepatitis (20.7%) (Table 1).

**TABLE 1:** Baseline characteristics of the study sample.

Variable	L (*M* < 1%) *N* = 1770	I (1% ≤ M < 5.76%) *N* = 5914	H (*M* ≥ 5.76%) *N* = 4450	*p*‐value
Age, mean ± SD	46.6 ± 23.1	54.3 ± 13.7	51.8 ± 16.6	< 0.01
Female, %	36	36.9	37.1	0.728
Living donor	0.2	0.4	0.2	0.310
LT indication, %				
Biliary atresia	8.4	1.1	1.7	< 0.01
Chronic viral hepatitis	15.9	19.4	22.2	< 0.01
ETOH cirrhosis	27.6	30.9	34.9	< 0.01
MASH	21.3	23.2	17	< 0.01
Acute liver failure	23.4	15.2	20.9	< 0.01
Autoimmune	3.4	5.1	6.7	< 0.01
Biliary cirrhosis	6.1	4.1	5.8	< 0.01
Primary hepatic malignancy	23.1	30.6	24.9	< 0.01
Liver metastasis	0.5	0.3	0.5	0.114
Income quartile, %				< 0.01
Q1	20.9	24.2	18.2	
Q2	26.9	27.7	25	
Q3	29.5	23.6	29.6	
Q4	22.7	24.4	27.2	
Coverage, %				< 0.01
Medicare	27.4	29.4	27.1	
Medicaid	18.3	11.3	16.6	
Private insurance	49.8	50	50.8	
Self‐pay	0.6	1.1	2	
Other	3.9	8.2	3.5	
Comorbidities, %				
MI	2.1	3	4.4	0.001
CHF	9.5	9.1	9.3	0.844
PVD	6.7	4.5	6.4	< 0.01
CVA	6.7	3.2	5.4	< 0.01
Dementia	0.6	0.1	0.6	< 0.01
COPD	13.5	13	12	0.198
Autoimmune	2.3	2.2	2.8	0.092
PUD	2.3	3.5	4	0.002
DM	17.1	20	19.3	0.028
HTN	0.7	1	1.1	0.247
CKD	28.5	29.5	33.5	< 0.01
AIDS	0	0.1	0.3	0.001
Obesity	13.3	16.4	14.5	< 0.01
Malnutrition	33.6	38.9	33	< 0.01
Frail (mFI ≥ 0.2)	45.5	51.3	48.5	< 0.01
CCI, median [IQR]	4 [2–5]	4 [2–5]	4 [2–6]	0.32
Hospital volume, %				< 0.01
Low	20.2	0	4.8	
Medium	55.8	7.4	23.3	
High	24	92.6	72	
Teaching status, %				< 0.01
Metrop nonteach	24.2	5.6	20.7	
Metrop teach	71.7	94.4	78.9	
Non‐metrop	4.2	0	0.4	
Disposition, %				< 0.01
Routine	71	41.4	37.6	
Short‐term hospital	1.5	1.3	2.5	
SNF	8.3	13.6	13.3	
Home health care	19.2	41	40.6	

The overall rate of FTR was 5%, with the most frequently observed complications being renal failure (60.6%) and respiratory failure (43.1%). Significant differences in FTR rates were observed among the center terciles (high risk: 8.8%, intermediate risk: 3.6%, low risk: 1.3%; *p* < 0.01). Multivariable logistic regression identified several independent predictors of FTR. Patient‐related predictors included low‐income quartile (OR 1.23, 95% CI: 1.11–1.39), malnutrition (OR 1.22, 95% CI: 1.09–1.29), biliary atresia as the presenting diagnosis (OR 3.39, 95% CI: 1.95–5.93), acute liver failure (OR 5.01, 95% CI: 4.09–6.15), CCI (OR 1.24, 95% CI: 1.18–1.31), and frailty (OR 1.58, 95% CI: 1.46–1.73) (Table 2).

**TABLE 2:** Incidence of postoperative complications.

Complication, %	L (*M* < 1%) *N* = 1770	I (1% ≤ M < 5.76%) *N* = 5914	H (*M* ≥ 5.76%) *N* = 4450	*p*‐value
Pulmonary				
Pneumonia	16.2	17.6	18.4	0.123
Respiratory failure	45.6	37.7	49.2	< 0.01
MV > 96 h	11	7.7	16.9	< 0.01
Tracheostomy	2.8	3	5.4	< 0.01
Cardiac				
Cardiac arrhythmia	19.1	18.7	21.7	0.001
Acute CHF	6.1	6.2	7.9	0.001
Cardiac ischemia	3.3	2.9	6.9	< 0.01
Infectious				
Sepsis	18	16.5	25	< 0.01
Abscess	5.5	4.2	6.8	< 0.01
C. diff	0.3	0.6	0.6	0.240
Meningitis	0	0.1	0.2	0.059
Aspergillosis	0.8	0.9	1.2	0.191
UTI	14.7	13.7	18	< 0.01
Candidiasis	9.4	6.6	9.1	< 0.01
Neurologic				
CVA	2	1.2	1	0.009
Hematologic				
VTE	3.5	3.9	6.4	< 0.01
Post‐op bleeding	9.3	8.8	13.4	< 0.01
GI bleed	10.4	8.4	14	< 0.01
Miscellaneous				
SBO	8.7	6.7	8.8	< 0.01
Shock	2.7	5.7	9.2	< 0.01
AKI	55.5	57	67.4	< 0.01
Functional dependence	5.1	7.5	6.6	0.003
Bile leak	0.3	0.4	0.5	0.592
Rejection	12.4	11.5	16.1	< 0.01
Graft dysfunction	3.7	2.3	5.2	< 0.01
Bowel perforation	0.8	0.8	0.5	0.135
Vascular complications	12.5	13.6	18.1	< 0.01
Wound dehiscence	4.2	3.1	5.7	< 0.01

Abbreviations: AKI: acute kidney injury; MV: mechanical ventilation; SBO: small bowel obstruction; UTI: urinary tract infection.

The univariate analysis demonstrated significant variations based on center mortality terciles. Patients at centers with the highest average mortality rates (H) had the highest 6‐month readmission rate (52.3%) compared to those at intermediate (1%–5.76%, I; 48.2%) and low (< 1%, L; 44.4%) mortality centers (*p* < 0.01). The median length of stay (LOS) was also the longest in the H group (15 days, interquartile range [IQR]: 8–30), followed by L (13 days, IQR: 9–27) and I (11 days, IQR: 7–22) (*p* < 0.01). The occurrence of any postoperative complication was significantly higher at high‐mortality centers (89%) compared to intermediate (82.1%) and low‐mortality centers (83.7%) (*p* < 0.01). Mortality rates increased markedly across the terciles, from 0.6% at low‐mortality centers to 3.8% at intermediate and 9% at high‐mortality centers. Similarly, FTR rates were significantly higher at high‐mortality centers (18.8%) compared to intermediate (7.7%) and low‐mortality centers (1.3%) (*p* < 0.01) (Table 3).

**TABLE 3:** Univariate analysis of outcomes.

Complication	L (*M* < 1%) *N* = 1770	I (1% ≤ M < 5.76%) *N* = 5914	H (*M* ≥ 5.76%) *N* = 4450	*p*‐value
6‐month readmission, %	44.4	48.2	52.3	< 0.01
LOS, days [IQR]	13 [9–27]	11 [7–22]	15 [8–30]	< 0.01
Any complication, %	83.7	82.1	89	< 0.01
Mortality, %	0.6	3.8	9	
FTR, %	1.3	7.7	18.8	< 0.01

Table 4 further highlights the distribution of complications and their respective FTR rates across center mortality terciles. High‐mortality centers had significantly greater rates of severe postoperative complications such as respiratory failure (49.66% incidence, 14.8% FTR), sepsis (25.19% incidence, 19% FTR), and cardiac ischemia (7.01% incidence, 54.3% FTR) compared to intermediate‐ and low‐mortality centers (*p* < 0.01). Notably, conditions such as bowel perforation and meningitis had the highest FTR rates among all complications at high‐mortality centers (62% and 44.6%, respectively). Mechanical ventilation for over 96 h was associated with an FTR rate of 31.7% at high‐mortality centers, compared to 21.6% at intermediate centers and 0% at low‐mortality centers. Similarly, tracheostomy was associated with a markedly increased FTR rate at high‐mortality centers (43.6%) compared to intermediate centers (21.1%). These findings highlight the differences between complication rates and FTR rates at high‐, intermediate‐, and low‐mortality centers (Table 4).

**TABLE 4:** Incidence of complications and FTR.

Complication	L (*M* < 1%)		I (1% ≤ M < 5.76%)		H (*M* ≥ 5.76%)		
	*N* = 1771		*N* = 5914		*N* = 4410		
	% C	% FTR	% C	% FTR	% C	% FTR	*p*‐value
Pneumonia	16.21	0	17.59	10.9	18.55	12	0.01
Respiratory failure	45.62	0	37.66	8.3	49.66	14.8	0.001
Cardiac arrhythmia	19.14	0	18.70	10.5	21.88	24	0.001
Acute CHF	6.10	0	6.17	9.8	7.94	21.4	0.001
Cardiac ischemia	3.27	0	2.93	21.2	7.01	54.3	0.0001
Sepsis	18.01	0	16.49	16.3	25.19	19	0.0001
C. diff	0.28	0	0.56	0	0.63	14.2	0.0001
Wound dehiscence	4.18	0	3.11	5.8	5.76	11.8	0.0001
CVA	1.98	0	1.18	22.2	1.04	26.6	0.009
VTE	3.50	0	3.92	6.7	6.42	22.2	0.0001
Postop bleeding	9.32	0	8.81	3.6	13.47	14.9	0.0001
SBO	8.70	0	6.68	1.1	8.91	20.7	0.0001
Abscess	5.48	0	4.23	4.9	6.85	22.9	0.0001
Shock	2.71	0	5.72	8.5	9.27	22.6	0.0001
AKI	55.51	0	57.34	5.8	68.03	11.4	0.0001
MV > 96 h	11.01	0	7.73	21.6	16.71	31.7	0.0001
Tracheostomy	2.77	0	3.03	21.1	5.42	43.6	0.0001
Functional dependence	5.14	0	7.46	3.7	6.69	4.5	0.003
Bile leak	0.34	0	0.36	0	0.48	0	0.592
Rejection	12.42	0	11.55	2.2	16.26	7.1	0.0001
GI bleed	10.39	0	8.42	11.5	14.13	21.6	0.0001
Graft dysfunction	3.67	0	2.33	16.7	5.24	45.7	0.0001
Bowel perforation	0.79	0	0.81	30.9	0.50	62	0.0001
Meningitis	0.00	0	0.10	0	0.23	44.6	0.0001
Aspergillosis	0.79	0	0.88	29.6	1.20	39.1	0.001
UTI	14.68	0	13.73	5.6	18.12	6.1	0.0001
Candidiasis	9.43	0	6.58	2.4	9.18	12	0.0001
Vascular complications	12.48	0	13.56	5.8	18.23	13.4	0.0001

Center‐related predictors included LT at a low‐volume center (< 20 cases/year) (OR 1.83, 95% CI: 1.78–2.01) and readmission to a different hospital (OR 2.08, 95% CI: 1.78–2.11). Conversely, protective factors against FTR were LT at a metropolitan teaching hospital (OR 0.96, 95% CI: 0.87–0.99), primary hepatic malignancy as a presenting diagnosis (OR 0.66, 95% CI: 0.52–0.86), high‐income quartile (OR 0.74, 95% CI: 0.57–0.96), discharge to a rehabilitation facility (OR 0.09, 95% CI: 0.03–0.26), and transplantation at a high‐volume center (> 50 cases/year) (OR 0.32, 95% CI: 0.20–0.49) (Table 5).

**TABLE 5:** Multivariable regression analysis: predictors of FTR.

Predictor	aOR	95% CI	*p*‐value
Low‐income quartile	1.23	1.11–1.39	0.01
Malnutrition	1.22	1.09–1.29	0.01
Biliary atresia	3.39	1.95–5.93	0.01
Acute liver failure	5.01	4.09–6.15	0.01
CCI	1.24	1.18–1.31	0.01
Frailty	1.58	1.46–1.73	0.01
Low‐volume center	1.83	1.78–2.01	0.01
Nonindex readmission	2.08	1.78–2.11	0.01
Metropolitan teaching hospital	0.96	0.87–0.99	0.01
Primary hepatic malignancy	0.66	0.52–0.86	0.01
High‐income quartile	0.74	0.57–0.96	0.01
Disposition to rehabilitation	0.09	0.03–0.26	0.01

Abbreviations: CCI: Charlson Comorbidity Index.

## 4 Discussion

Our study highlights substantial variability in FTR rates among LT centers and demonstrates the influence of both patient‐ and center‐related factors on postoperative outcomes. High‐volume and metropolitan teaching hospitals demonstrated significantly lower FTR rates, suggesting that institutional expertise, resource availability, and care coordination contribute to improved rescue rates. Conversely, patients treated at low‐volume centers and those requiring interhospital transfers faced heightened risks, emphasizing the need for standardized care protocols and enhanced post‐transplant surveillance strategies.

Our data show lower income, malnutrition, biliary atresia, acute liver failure, CCI, frailty, lower‐volume centers, and nonindex readmission to be predictors of FTR. Of these, several could be considered modifiable, including frailty and socioeconomic status. Additionally, receiving care at a metropolitan teaching hospital, having primary hepatic malignancy, having a high‐income quartile, and having a disposition to rehabilitation were all protective against FTR. These findings suggest that targeted prehabilitation, nutritional optimization, and social support interventions could improve postoperative outcomes and, in turn, decrease rates of FTR [12]. Further, understanding the relationship between these factors and FTR can inform and improve care even with factors that are not modifiable.

The concept of frailty is relatively new when it comes to LT. Several studies in recent times have consistently demonstrated that frailty negatively affects liver transplant outcomes, including hospitalizations and mortality [14, 15]. Recent efforts have been made to standardize frailty measurement in LT by introducing the Liver Frailty Index, which has been developed to assess frailty and physical function in this population [16]. The Liver Frailty Index was developed specifically to measure the construct of physical frailty in liver transplant candidates through handgrip strength, chair stands, and balance testing. It is a highly predictive indicator of waitlist mortality in liver transplant candidates [16].

Our study showed higher rates of FTR in low‐volume centers. Over the last 2 decades, a strong relationship has been reported between higher volume and better outcomes. For example, in a study by Axelrod et al., unadjusted 1‐year mortality rates for liver transplants were significantly different at high‐ (15.9%) versus low‐ (16.9%) or medium‐ (14.7%) volume centers [17]. After adjustment, low‐volume centers were associated with a significantly higher risk of death.

It is crucial to differentiate between broadly improving postoperative outcomes and specifically reducing FTR rates. FTR reflects not only patient survival but also a hospital's ability to promptly recognize and manage complications [4]. FTR was first identified by Silber et al., who concluded that FTR was more strongly associated with hospital factors than with patient severity of illness at admission [2]. Hospital characteristics described in this article, such as size and type of hospital, contribute to FTR. However, these factors alone do not account for all the variability in FTR rates among hospitals. Factors on a smaller scale, such as institutional safety culture, teamwork, interprofessional communication, and other health care personnel attitudes and behaviors, may interact with the hospital's physical and technological resources to effectively manage postoperative complications and prevent FTR events [18, 19]. These may inhibit steps in the process of clinical rescue by acting as barriers to initiating appropriate escalation of care or by contributing to ineffective team communication [19].

The Pareto principle, which suggests that 20% of inputs drive 80% of outcomes in complex systems, is particularly relevant in this context [20]. Research has shown that approximately 20% of patients at the highest risk for postoperative complications account for nearly 90% of FTR cases [21]. This is an example of the Pareto principle as it applies to the complex process of postoperative complications and subsequent death. Although originally an economic concept, its relevance to medicine suggests that targeting interventions toward this high‐risk subset of patients could yield the most significant reduction in FTR rates.

Using data from the NRD, we analyzed FTR in LT, but the database has limitations. First, there are limited data on complications, which means that despite including several important categories of adverse postoperative outcomes, there is still a possibility of missing some important complications. Furthermore, this analysis does not provide data on the cause of death, so it is not clear whether these complications directly influenced mortality. Because of the lack of details in the database, we cannot determine which factors can be modified to improve FTR rates. Moreover, MELD scores were not available in the NRD, limiting our ability to adjust for baseline liver disease severity and potentially introducing residual confounding. In addition, our study identified several factors associated with FTR. However, some factors are often patient‐related and cannot always be changed. We cannot overemphasize how important this limitation is because the real value of analyses like ours lies in the guidance they provide for implementing change. The current analysis presents a framework for bringing FTR considerations to the field; however, much more detailed consideration is needed before FTR is ready for clinical application. To routinely use FTR in LT for quality improvement, a sophisticated understanding of factors affecting patient and center outcomes is necessary. Future research should focus on implementing evidence‐based protocols to reduce FTR in LT. Prospective studies evaluating the impact of structured perioperative programs, such as enhanced recovery pathways and telemedicine follow‐up visits, may further identify avenues for quality improvement in LT outcomes.

## 5 Conclusion

FTR remains a critical issue in LT, with significant variability across centers. These findings demonstrate associations, not causation, between center‐ and patient‐level factors and FTR rates. Identifying and addressing modifiable predictors of FTR presents opportunities for improving perioperative management and postoperative care.

## Author Contributions

**Kamil Hanna:** conceptualization, methodology, writing – original draft, writing – review and editing. **Peyton Seda:** conceptualization, methodology, writing – original draft, writing – review and editing. **Brian C. Longbottom:** conceptualization, methodology, writing – original draft, writing – review and editing. **Shengliang He:** formal analysis, data curation, validation, writing – review and editing. **Inkyu Lee:** formal analysis, data curation, visualization, writing – review and Editing. **Avery Wilson:** formal analysis, validation, writing – review and Editing. **Kenji Okumura:** formal analysis, data curation, validation, writing – review and editing. **David Axelrod:** investigation, validation, writing – review and editing. **Hassan Aziz:** supervision, project administration, resources, writing – review and editing.

## Conflicts of Interest

The authors declare no conflicts of interest.

## Data Availability

The data that support the findings of this study are available in the supplementary material of this article.
